# Calcineurin Undergoes a Conformational Switch Evoked via Peptidyl-Prolyl Isomerization

**DOI:** 10.1371/journal.pone.0134569

**Published:** 2015-08-06

**Authors:** Alicia Guasch, Álvaro Aranguren-Ibáñez, Rosa Pérez-Luque, David Aparicio, Sergio Martínez-Høyer, M. Carmen Mulero, Eva Serrano-Candelas, Mercè Pérez-Riba, Ignacio Fita

**Affiliations:** 1 Institut de Biologia Molecular de Barcelona (IBMB-CSIC), Parc Científic, Baldiri Reixac 10, 08028, Barcelona, Spain; 2 Human Molecular Genetics Laboratory, Institut d’Investigació Biomèdica de Bellvitge (IDIBELL), Gran Via de L’Hospitalet 199, L’Hospitalet de Llobregat, 08908, Barcelona, Spain; NCI-Frederick, UNITED STATES

## Abstract

A limited repertoire of PPP family of serine/threonine phosphatases with a highly conserved catalytic domain acts on thousands of protein targets to orchestrate myriad central biological roles. A major structural reorganization of human calcineurin, a ubiquitous Ser/Thr PPP regulated by calcium and calmodulin and targeted by immunosuppressant drugs cyclosporin A and FK506, is unveiled here. The new conformation involves *trans-* to *cis-* isomerization of proline in the SAPNY sequence, highly conserved across PPPs, and remodels the main regulatory site where NFATc transcription factors bind. Transitions between *cis-* and *trans-* conformations may involve peptidyl prolyl isomerases such as cyclophilin A and FKBP12, which are known to physically interact with and modulate calcineurin even in the absence of immunosuppressant drugs. Alternative conformations in PPPs provide a new perspective on interactions with substrates and other protein partners and may foster development of more specific inhibitors as drug candidates.

## Introduction

Reversible phosphorylation, orchestrated by the opposing activities of kinases and phosphatases, is estimated to occur in about one-third of proteins and is responsible for modulating many cellular functions including cell growth, proliferation and differentiation [1, 2]. Phosphoserine and phosphothreonine, which account for over 98% of protein-bound phosphate in eukaryotic cells, are regulated by a large number of Ser/Thr protein kinases and, surprisingly, far fewer Ser/Thr phosphatases including, in particular, the widely distributed family of phosphoprotein phosphatases (PPPs), encoded by a relatively small number of genes in the human genome[3]. Protein phosphatase 1 (PP1), with more than two hundred confirmed targeting proteins, and calcineurin (PPP3C, formerly PP2B) are the most abundant and extensively studied PPPs [4].

**Fig 1:**
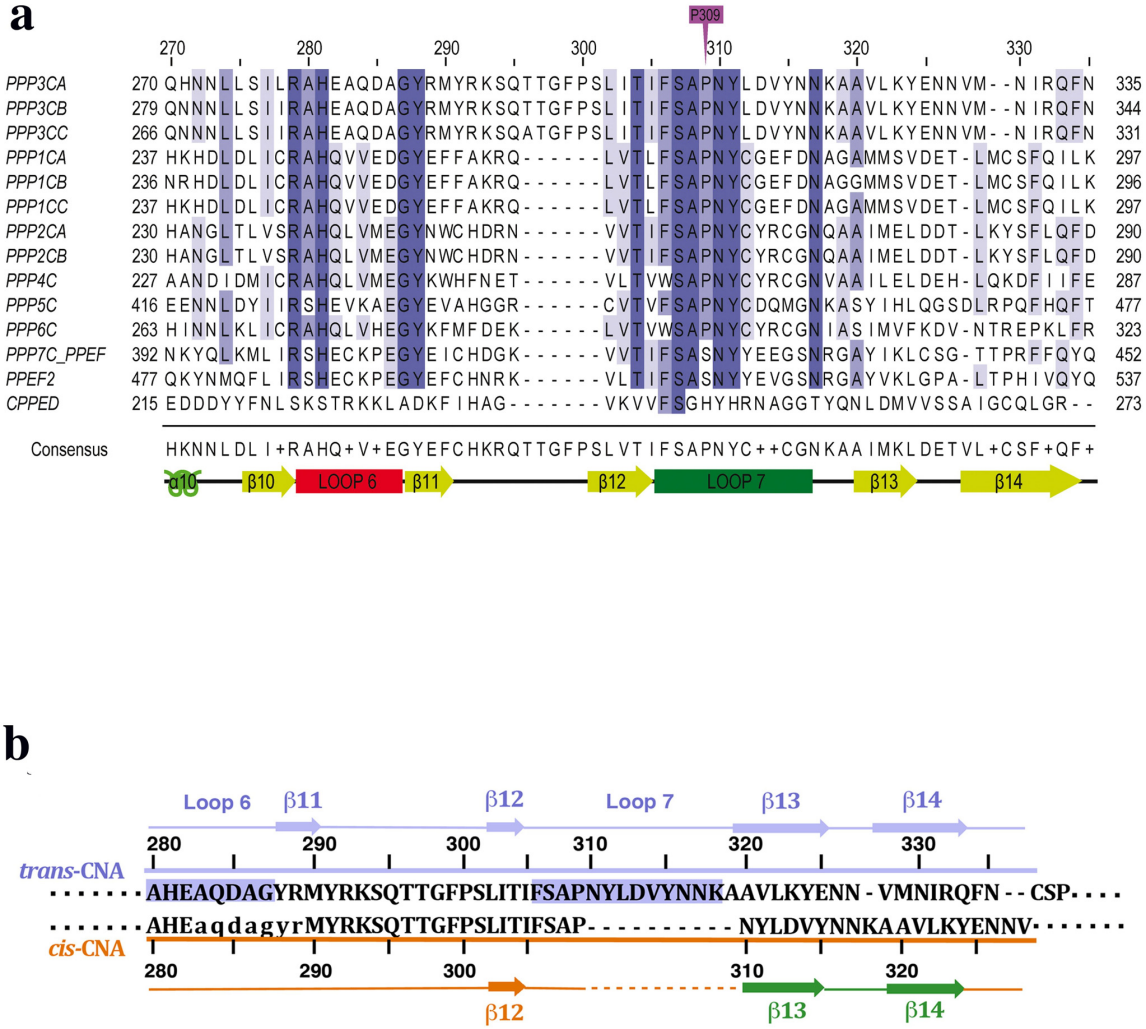
Sequence and structural alignments. (**a**) The catalytic domain of CNA is highly conserved around the β12 and β13 connection in PPPs. Sequence alignments around the β12 and β13 connection of the catalytic domains of human PPPs. PPP1 to PPP7, protein phosphatases 1 to 7; PPEF1 and 2, protein phosphatase EF-hand calcium binding domain 1 and 2; and CPPED1, calcineurin-like phosphoesterase domain containing 1. A, B and C correspond to α, β and γ isozymes, respectively. Alignment were performed using MAFFT v.7 online version (http://mafft.cbrc.jp/alignment/software/)[38] using default parameters and subsequently edited using Jalview software v.2.8[39]. (**b**) Structural alignment of *cis*-CNA (brown) and *trans*-CNA (blue) showing the sequence shifts and the secondary structural elements correspondence. Small letters indicate amino acid residues not modeled in the structure. Residues from loops 6 and 7 are shadowed in the *trans*-CNA conformation.

Calcineurin (CN), ubiquitously expressed and highly conserved from yeast to humans, plays a critical role coupling Ca^2+^ signals to different gene expression patterns and cellular responses[5]. CN was first identified as the target of the immunosuppressants cyclosporine A (CsA) and FK506[6, 7], which are the cornerstone of current immunosuppressive therapy. These drugs bind to the endogenous immunophilins cyclophilin A (CypA) and FKBP12[8], respectively, and the corresponding complexes bind to CN inhibiting phosphatase activity for all CN substrates resulting in both desired therapeutic outcomes and in some cases severe side-effects[9]. CN functions as an heterodimer with a large catalytic subunit (CNA) (59 kDa for the human α isoform) interacting with two Ca^2+^ binding proteins, calmodulin and the small regulatory CN subunit (CNB) (19 kDa for the type 1 isoform)[5]. CNA is organized as an N-terminal catalytic domain, of about three hundred residues, and a C-terminal regulatory domain comprising the CNB and the calmodulin binding regions followed by an autoinhibitory domain (AID) that, under specific conditions, binds into the active site blocking the access of substrates [10]. The catalytic domain of CNA presents a high sequence and structural homology to other PPP catalytic domains [11, 12]. The active site contains two metal ions located between two helical domains and a characteristic central sandwich of two β-sheets that are interconnected between strands β12 (from sheet I) and β13 (from sheet II) by a highly conserved sequence (FSAPNYxxxxxNx) (Fig 1a)[13]. The β12-β13 connection, also called loop 7 in CNA, is one of the key regulatory elements in PPPs [14, 15].

The cytosolic Nuclear Factor of Activated T cells (NFATc) transcription factors, critical in many cellular processes, bind to CN through two binding sequences, the PxIxIT and LxVP motifs[16, 17], and at least one of them is present in all the endogenous CN modulators identified so far[18]. The structures of a diversity of CN constructs alone or in complex with FK506/FKBP12[11, 12], CsA/CypA[19, 20], or with different peptides, have been determined[18, 21–23]. Regulatory PxIxIT-containing peptides add a strand at the β14 edge of β-sheet II. Similarly, PP1 phosphatase recognizes an RVxF motif in its targeting subunits through binding at a site cognate to the PxIxIT-binding site [1, 24].

## Materials and Methods

### Materials

Peptides were purchased from the Peptide 2.0 Company (Chantilly, VA) and synthesized as acetylated N-terminal and C-terminal amides for the unlabeled peptides and N-carboxyfluorescein (CF) and C-terminal amide for the labelled peptide. The sequences of the peptides used are the following: KYELHAGTESTPSVVVHVCES for the RCAN^183–203^ peptide; NNKAAVLKYE for the *cis*-CNA derived peptide; and ASGLSPRIEITPSHEL for the NFATc2-SPRIEIT (SPRIEIT) peptide. All peptides were resuspended in 100% DMSO at 10 mM. Cyclosporin A (CsA) was obtained from Sandoz. Ionomycin (Io) sodium salt and Phorbol 12-myristate 13-acetate sodium salt (PMA) were obtained from Sigma. The anti-calcineurin A antibody was purchased from BD Biosciences and the anti-Flag M2 antibody was purchased from Sigma. The protease and phosphatase inhibitor cocktails were from Calbiochem.

### Methods

#### Protein expression and purification

Large scale production of the catalytic domain of CNA was achieved with the pGEX-6P-1-CNAα plasmid construct, kindly provided by Patrick Hogan, that encodes the Glutathione S-transferase protein linked to the human CNA α isoform (NCBI NP_000935.1) catalytic domain (residues 2–347). Expression, purification and isolation of the CNAα domain was performed as previously described[25]. The isolated CNAα catalytic domain was further purified using a Superdex 75 size-exclusion column equilibrated at 0.4 ml/min with 100 mM NaCl and 50 mM Tris pH 8.0, where the protein eluted as a unique peak corresponding to 40 kDa. The purified protein was kept at 4°C for short periods of time.

#### Protein crystallization and structure determination

Hexagonal crystals were obtained by vapour diffusion, using the human CNAα catalytic domain a final protein concentration of 7.3 mg/ml. Crystals (with a reservoir buffer of 0.1M Hepes—pH 7.5 -, 26% PEG 3350 and 4% PGA) are in space group P6_2_22 with cell parameters of **a** = **b** = 185.01 Å, **c** = 106.74 Å and **α** = **β** = 90°, **γ** = 120°. Crystals were cryoprotected using reservoir solution supplemented with 20% (v/v) glycerol and flash-cooled in liquid nitrogen. X-ray diffraction data, collected at 100K on beam line PROXIMA-1 (SOLEIL synchrotron, France) with a Dectris Pilatus 6M detector and oscillation angles of 0.2° per frame, were processed using the interactive iMOSFLM package at 3.35 Å resolution[26]. Crystals, with similar unit cell parameters, have also been obtained in the absence of inhibitory peptides, though diffraction from these crystals was always below 3.5 Å resolution.

The structure was solved by molecular replacement using *MOLREP*[27] and the coordinates of human CN (PDB entry 1AUI) as search model. Refinement was performed with *REFMAC5*[28] and the molecular-graphics program *COOT*[29] (Table 1). Non-crystallographic restraints together with the restraints of the *trans*-CNA subunit towards high resolution information were also applied.

**Table 1:** X-ray Data and Refinement statistics.

**A**	
**X-ray Data**	CNA
Resolution limits (Å)	50.0–3.35 (3.53–3.35)
Space group	P6_2_ 2 2
Unit cell parameters (Å, °)	185.01, 185.01, 106.74 90, 90, 120
R pim (%)	5.2 (33.6)
Completeness (%)	100 (100)
<I/σ(I)>	13.0 (2.7)
Multiplicity	17.1 (15.7)
N° of unique reflections	15.994 (2.270)
Total number of observations	272.976 (33.646)
**B**	
**Refinement**	CNA
Resolution limits (Å)	50.0–3.35
Rwork (%)	21.56
Rfree (%)	24.91
Rms bond lengths (Å)	0.017
Rms bond angles (°)	2.1
N° of protein atoms	5.064
Protein mean B-factor (Å^2^)	48

* In brackets for the last resolution shell.

#### Fluorescence polarization

Competition assays were performed as described[30]. Briefly, carboxyfluorescein (CF)-SPRIEIT-CNA complex was performed using 10 nM of CF-labeled SPRIEIT peptide and 10 μM CNA. Unlabeled competitor peptides were pre-incubated with CNA at increasing concentrations for 15 min before adding the fluorescence labeled peptide. Experiments were performed in a OptiPlate black 384-well-flat-bottom plates (PerkinElmer Life Sciences, Waltham, MA) and measured using a Wallac VICTOR (TM) X5 2030 Multilabel Reader (PerkinElmer Life Sciences) with excitation and emission wavelengths of 485 nm and 535 nm respectively. All assays were performed for 15 min at room temperature. All data were obtained from at least three independent experiments performed in triplicates.

#### GST-pull-down competition assays

In GST-RCAN3 pull-down competition assays, HEK 293T cells were lysed in co-immunoprecipitation buffer (50 mM Tris–HCl, pH 7.5, 100 mM NaCl, 2 mM CaCl_2_, 5 mM MgCl_2_, 1% IGEPAL, 1 mM DTT, 2 mM PMSF and protease and phosphatase inhibitor cocktails) and the CNA- and the RCAN3-derived peptides containing a PxIxIT sequence were added to the extracts at the indicated concentrations for 30 min. Then, the soluble extracts were incubated with GST–RCAN3 bound to Glutathione Sepharose beads for 90 min at 4°C. After extensive washing, co-precipitated proteins were eluted by resuspension in 2× Laemmli buffer and boiled for 10 min. For GST–NFATc2 pull-down competition assays, HEK 293T cells transfected with FLAG–CNA (2–389) were lysed in a buffer containing 50 mM Tris–HCl, pH 8, 100 mM NaCl, 1.5 mM CaCl_2_, 6 mM MgCl_2_, 0.2% TX-100, 1 mM PMSF and protease and phosphatase inhibitor cocktails. Soluble extracts were incubated with GST–NFATc2 bound to Glutathione Sepharose beads for 60 min at 4°C.

#### NFATc-luciferase reporter gene assay

Flag-hCNAα mutants were performed by PCR using CN specific primers (Table 2). ΔNIR mutant lacks the CNA PXIXIT binding region VMNIR ranging from amino acids 328 to 332. All Flag-CNA constructs bear the Y341F mutation to achieve CsA resistance unless for the specified wt. Luciferase gene assays were performed in HEK 293T cells transfected with 100 ng of 3xNFAT-luc plasmid, 100 ng of pBJ5-mCNB, 1 ng of pRLNull as an internal transfection control and 400 ng of the indicated Flag-hCNAα mutants constructs. Stimulation was achieved by treating the cells with.1 μM ionomycin, 10 nM PMA and 10 mM CaCl_2_. Endogenous CN activity was abolished by treating cells with 1 μM Cyclosporin A (CsA) 30 min prior to stimulation. FK506 was used as a positive control for CsA-resistant CN. After 6h of cell stimulation, 10 μl of cell extract were analyzed for luciferase gene expression using the Dual-Luciferase Reporter Assay (Promega) following the manufacturer’s protocol on a multiplate luminometer (FLUOstar Optima, BMG). Luciferase units were normalized to densitometric data of 10 μl of cell extract analyzed by western blot. Absence of cell stimulation is shown with a minus symbol (-). Three experiments were performed with triplicates. Stimulation of the Y341F mutant was considered as 100% value.

**Table 2:** Oligonucleotide designation and sequence.

Primer name	Sequence
hCnA-Y341F-Fw	GCAATTCAACTGTTCTCCTCATCCATTCTGGCTTCCAAATT
hCnA-Y341F-Rv	AATTTGGAAGCCAGAATGGATGAGGAGAACAGTTGAATTGC
hCnA-Y288N-Fw	CCCAGATGCAGGGAACCGCATGTACAGG
hCnA-Y288N-Rv	CCTGTACATGCGGTTCCCTGCATCTTGGG
hCnA-Y288F-Fw	GAAGCCCAAGATGCAGGGTTCCGCATGTAC
hCnA-Y288F-Rv	GTACATGCGGAACCCTGCATCTTGGGCTTC
hCnA-Y288A-Fw	GAAGCCCAAGATGCAGGGGCCCGCATGTACAGGAAAAG
hCnA-Y288A-Rv	CTTTTCCTGTACATGCGGGCCCCTGCATCTTGGGCTTC
hCnA-ΔNIR-Fw	CAGTATTGAAGTATGAGAACAATCAATTCAACTGTTCTCCTCATCC
hCnA-ΔNIR-Rv	GGATGAGGAGAACAGTTGAATTGATTGTTCTCATACTTCAATACTG

## Results and Discussion

**Fig 2:**
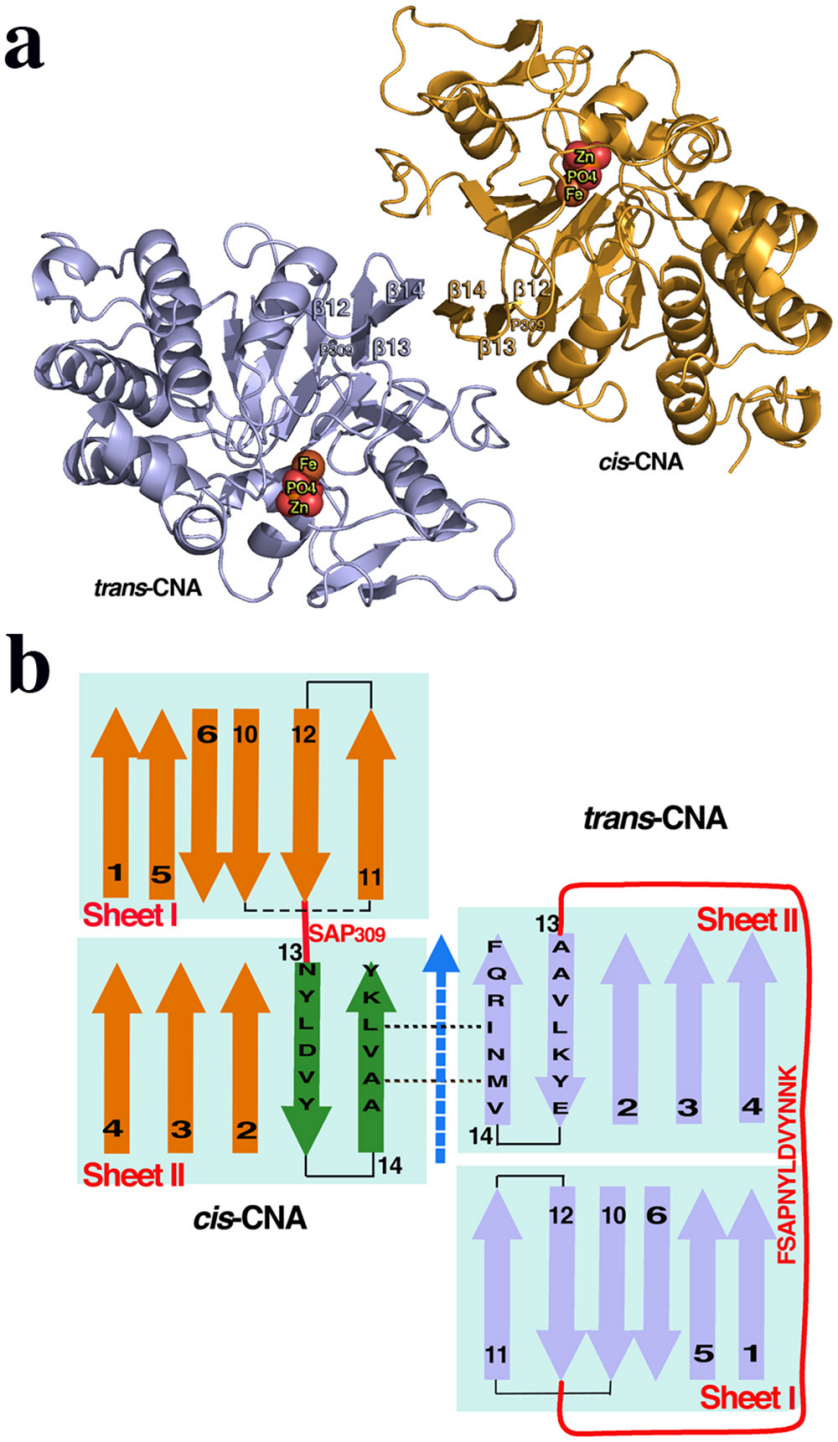
Structures of the interacting CNA catalytic domains with *trans-* and *cis-* conformations. (**a**) Ribbon representation of two neighboring CNA subunits in the crystal presenting the *trans-* (blue) and *cis-* (brown) conformations, respectively. The metal atoms and the phosphate group in the active site are shown as spheres. (**b**) Sheets I and II topology corresponding to the *cis*- and *trans*-conformations of the catalytic domain of CNA with the relative position of the two subunits in the crystal showing the interaction between β14 strands. The pseudo two-fold axis (dashed blue arrow) relating both subunits is indicated. β13 and β14 strands in *cis*-CNA are shown with a different color (green) to emphasize that they differ from the ones in *trans*-CNA. The thirteen residues long loop 7 joining strands β12 and β13 in *trans*-CNA is reduced to a tight β-turn with only four residues (SAP_309_N) in *cis-*CNA.

**Fig 3:**
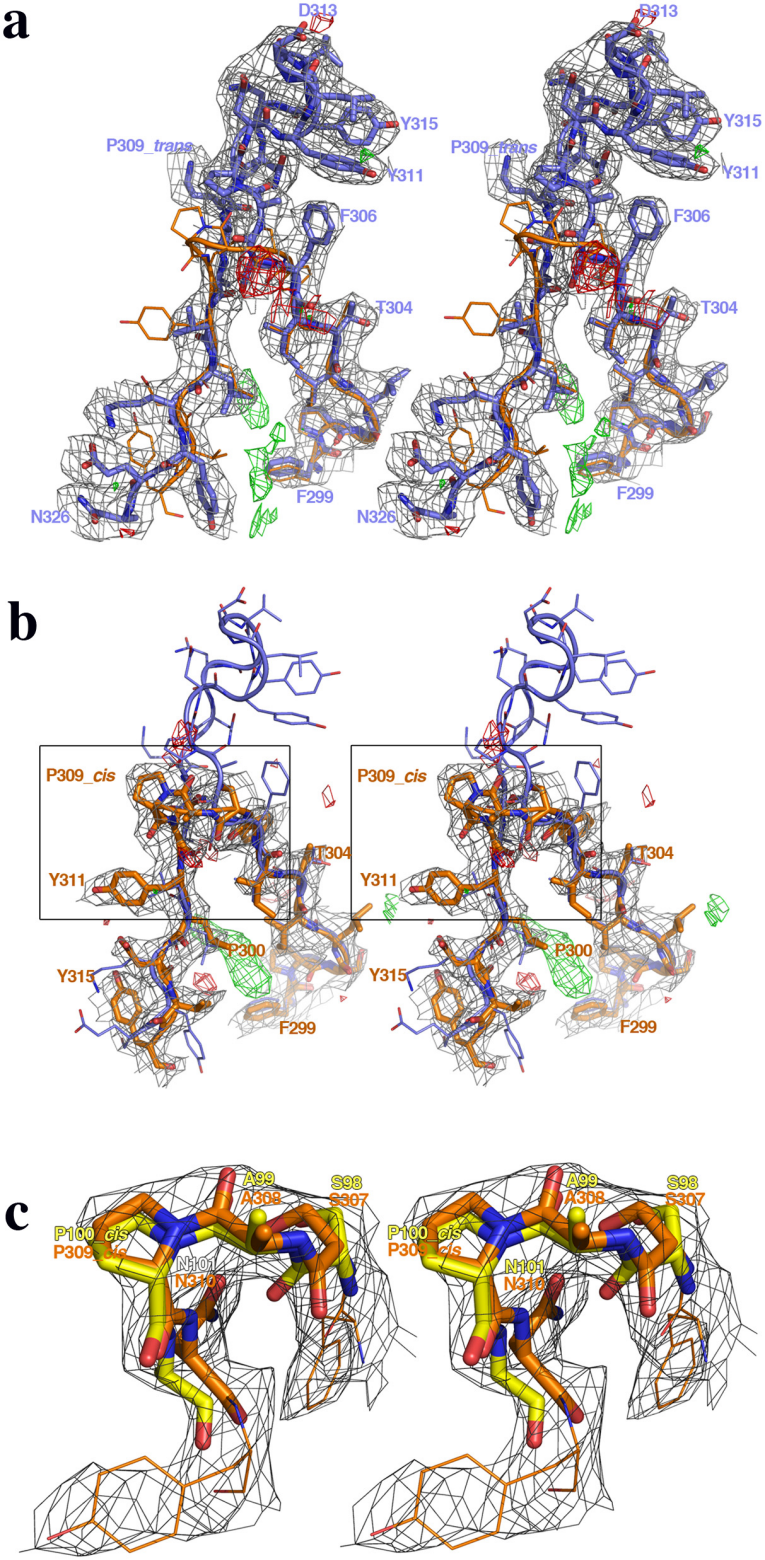
Complete reorganization of the Pro309 environment between the *trans-* and *cis-* conformations of CNA. Stereo views showing the large structural differences observed in the Pro309 environments between the (**a**) *trans*-CNA and (**b**) *cis*-CNA conformations. The (2Fo-Fc) electron density map, contoured at 1σ and depicted in dark grey, and the (Fo-Fc) maps, contoured at 3σ (positive in green and negative in red), are also shown for both CNA conformations. Residues from Phe299 to Asn326 and from Phe299 to Tyr315 are shown for *trans*-CNA (blue) and *cis*-CNA (brown), respectively. The corresponding molecular models are represented as thick rods while the superimposed structures of the alternative conformations are depicted as thin rods. Both conformations are essentially identical till residue Phe306, where only side chains differ significantly. Beyond Phe306 also main chains separate completely. (**c**) Superimposition of the structures of the-Ser-Ala-*cis*Pro-Asn- fragment forming the turn (within the SAPNY sequence), as found in *cis-*CNA (brown) and in the enzyme family 5 xyloglucanase (yellow) where the *cis* conformation adopted by the proline is accurately defined at 1.4 Å resolution (PDB code 2JEP). The 2Fo-Fc electron density corresponding to the boxes in panel **b** is also shown to emphasize the quality of the fitting of *cis-*CNA residues around Pro309 (from Phe306 to Tyr311).

The structure of the catalytic domain of human CNAα (residues 2–347) has been determined from hexagonal crystals. The two subunits of the catalytic domain contained in the asymmetric unit presenting important structural differences (Table 1, Fig 2). The first subunit in the crystal shows the essentially invariant conformation (herein referred to as *trans-*CNA) found in all available structures of CN and other PPP phosphatases. The second subunit in the crystal shows an alternative new conformation (herein referred to as *cis-*CNA) (Fig 2a). The two subunits in the asymmetric unit are related by a non-crystallographic quasi-two-fold rotation (172°) plus a 3.7Å translation, about an axis roughly in the middle of the β14 strands that interact with each other extending sheet II across both subunits (Fig 2b). The thirteen residues long connection (Phe306-Lys318) joining strands β12 and β13 in *trans*-CNA is reduced to a standard VIa-1 β-turn [31] with only four residues SAP_309_N in *cis-*CNA (Figs 1a, 1b and 2b). Residue Pro309 within the SAPNY sequence is a peptidyl *trans* isomer in *trans-*CNA, while it is a *cis* isomer in *cis-*CNA (Fig 3). Strands β13 and β14, downstream in sequence to Pro309, are still present in *cis-*CNA and superimpose structurally well with the same strands of *trans-*CNA, but the amino acid residues within these strands differ between *trans*-CNA and *cis*-CNA (Figs 1b, 3a and 3b). Strand β13 presents a ten residue shift between the two conformations with Asn310 in *cis*-CNA being structurally equivalent to Ala320 in *trans*-CNA. For the β14 strand the structurally equivalent residues present a nine residue shift with Ala319 in *cis*-CNA being structurally equivalent to Val328 in *trans*-CNA. Therefore, the nine residue shift increases the number of residues after β14 in *cis*-CNA with respect to *trans*-CNA. This region loops around the active site in *cis*-CNA, with side chains poorly defined, interacting also with neighboring subunits in the crystal. This region would correspond to the linker connecting the catalytic domain and the CNB binding region in full-length CNA. In *trans*-CNA, loop 7 and strand β13 interact with loop 6, which corresponds to residues from Ala280 to Gly287 between strands β10 and β11 including the catalytically essential residue His281[2] (Fig 1b). The decrease in the number of residues for the β12-β13 connection together with the amino acid sequence changes in strand β13, can explain the destabilization of loop 6 and of strand β11 that in *cis*-CNA are disordered from Ala283 until Arg289.

**Fig 4:**
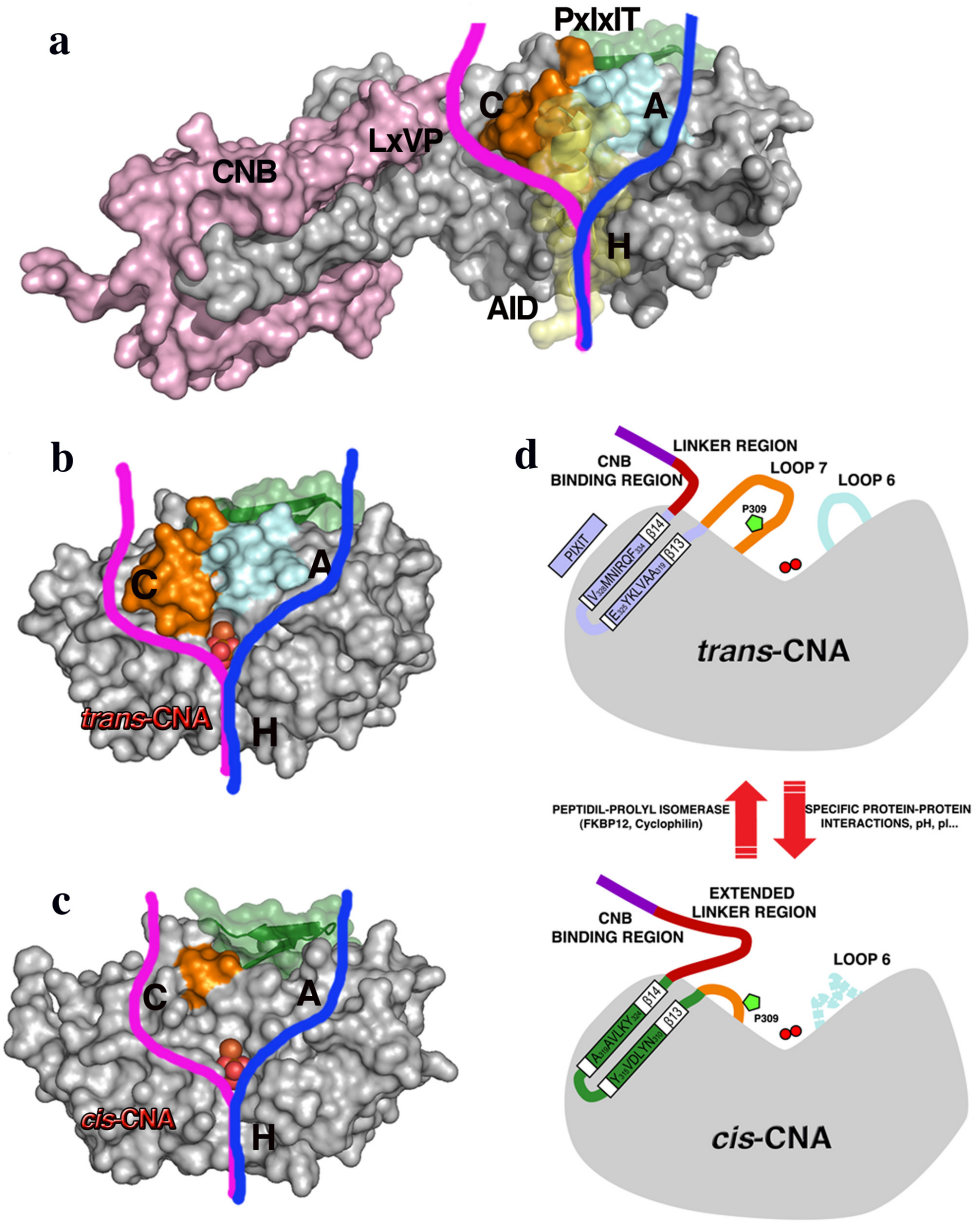
Reorganization of the active site between *trans*- and *cis-* CNA. (**a**) Surface representation of a CNA subunit (grey), truncated after the CNB binding region, in complex with a CNB subunit (pink), with the binding sites for proteins containing LxVP and for PxIxIT (green) motifs [18]. A bound autoinhibitory domain is also explicitly indicated (AID in yellow). The acidic, C-terminal and hydrophobic substrate-binding grooves, defined for PPP phosphatases, are labeled as A, C and H, respectively. Loops 6 (light blue) and 7 (brown) are clearly visible between the acidic and C-terminal grooves. Surface representation of the catalytic domain of CNA subunit illustrating the *trans*- conformation (**b**) and the *cis*- conformation (**c**). In these views the two catalytic metal ions and a bound phosphate molecule are clearly visible. The C-terminal tail of *cis*-CNA, corresponding to the extended linker, has been omitted for clarity. (d) Cartoon of the *cis-/trans-*CNA transition with a schematic representation of the main structural differences between the *trans*-CNA (upper) and *cis*-CNA (bottom) conformations. Strands β13-β14 are colored in light blue and green for the *trans*- and *cis-*CNA conformations, respectively. Metal ions (red balls), loop6 (light blue) and loop7 (brown), with proline 309 (green pentagon), are depicted.

**Fig 5:**
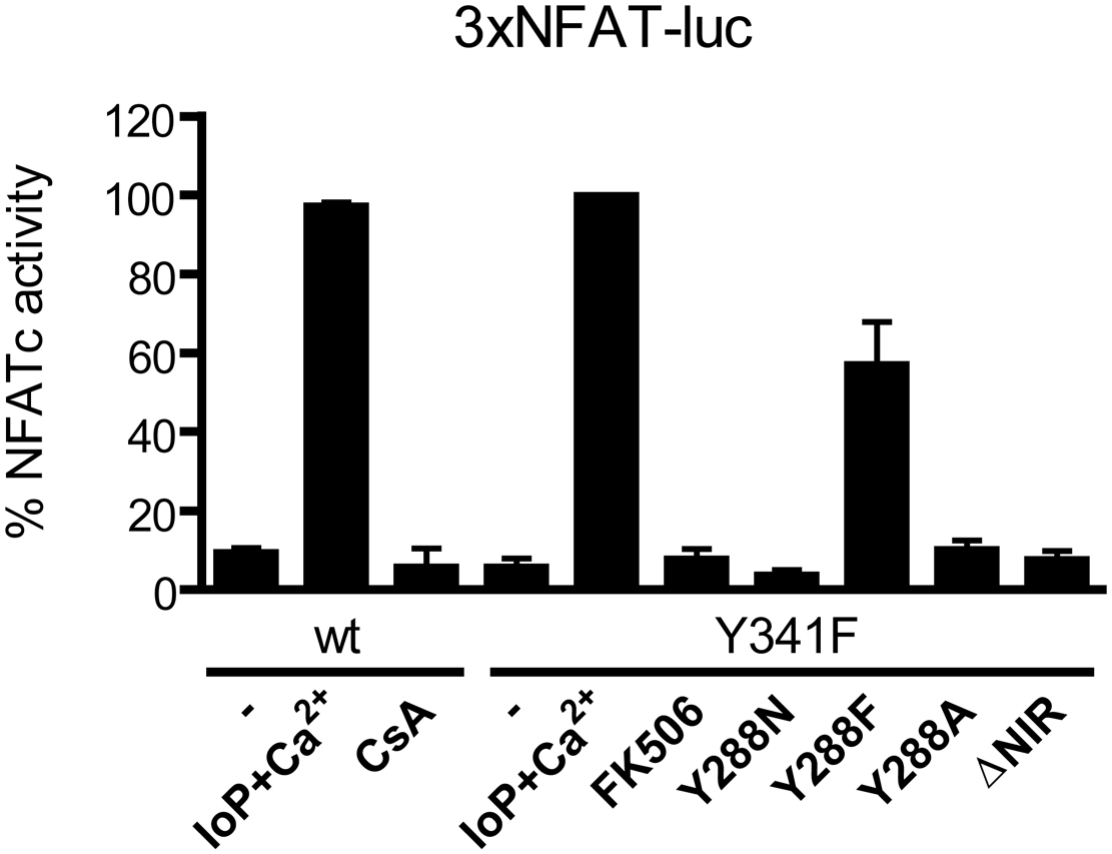
Mutation of loop 6 residues affects NFATc activity. Luciferase reporter gene assays in HEK 293T cells transfected with 3xNFAT-luc plasmid, pBJ5-mCNB and Flag-CNA 2–389 wild type (wt) or related mutants. ΔNIR mutant lacks the CNA PXIXIT binding region VMNIR ranging from amino acids 328 to 332. All Flag-CNA constructs bear the Y341F mutation to achieve CsA resistance unless for the specified wt. Endogenous CN activity was abolished by treating the cells with 1 μM CsA 30 min before stimulation. Stimulation was achieved by treating the cells with Io/PMA/Ca^2+^ for 6 hours. FK506 was added as a control of CN inhibition of the CNA 2–389 Y341F. Absence of cell stimulation is shown with a minus symbol (-). Three experiments were performed in triplicates. CNA wt and mutants protein levels were assessed by densitometry of the electrophoretic bands detected by western blot analysis with anti-FLAG antibody. Data is given as mean percentage of NFAT activation normalized to CNA protein levels. Stimulation of the Y341F mutant was considered as 100% value.

Differences between *trans*-CNA and *cis*-CNA have major implications both on the active site organization and on the crucial docking site for PxIxIT-containing proteins. Concerning the active site, PP1 phosphatases have been described as containing two metal ions at the intersection of three putative substrate binding grooves, referred to as hydrophobic, acidic and C-terminal grooves[24]. Following on this description for CN (Fig 4a), loops 6 and 7 are located between the acidic and the C-terminal grooves forming the internal walls of these grooves in *trans-*CNA (Fig 4b). Instead, in *cis*-CNA the acidic and C-terminal binding grooves merge into a continuous surface, due to the reduction of loop 7 and the flexibility of loop 6, rendering the catalytic metal ions accessible to substrates with wider binding surfaces than for *trans-*CNA (Fig 4c). Moreover, in *cis*-CNA disordered residues from loop 6 and the enlarged linker define a new environment around the active site (Fig 4d). Residues from loop 7 (in particular Tyr311 and Tyr315) in *trans*-CNA participate in AID binding and consequently the absence of this loop 7 in *cis*-CNA should weaken or prevent AID binding and the corresponding inhibitory effects. Given that in *cis*-CNA the active site is more accessible than in *trans*-CNA, no significant differences in phosphatase activity are expected for the standard small substrates pNPP and RII peptide (from the regulatory RII subunit of cAMP-dependent protein kinase[32]). Concerning the PxIxIT binding site, in *trans*-CNA strand β14 defines the central structural element of the site with binding affinities that are finely tuned from both consensus and non-consensus positions in the motif[18, 21]. Accordingly, affinity for the PxIxIT binding site is redefined by the sequence shift between β14 strands in *trans*-CNA and *cis*-CNA (Figs 1b and 2b). Mutating residue Tyr288, a residue next to loop 6 that is disordered in cis-CNA and in trans-CNA interacts with Lys323 from β-13 and with Ile331 from β-14, to alanine or to asparagine decreases sharply NFATc activity (Fig 5). In turn, mutant of Tyr288 to phenylalanine, which can retain the hydrophobic interactions with Ile331, also retains a significant NFATc activity. Therefore, altering loop 6 results in the destabilization of trans-CNA and consequently of the CN PxIxIT binding site.

**Fig 6:**
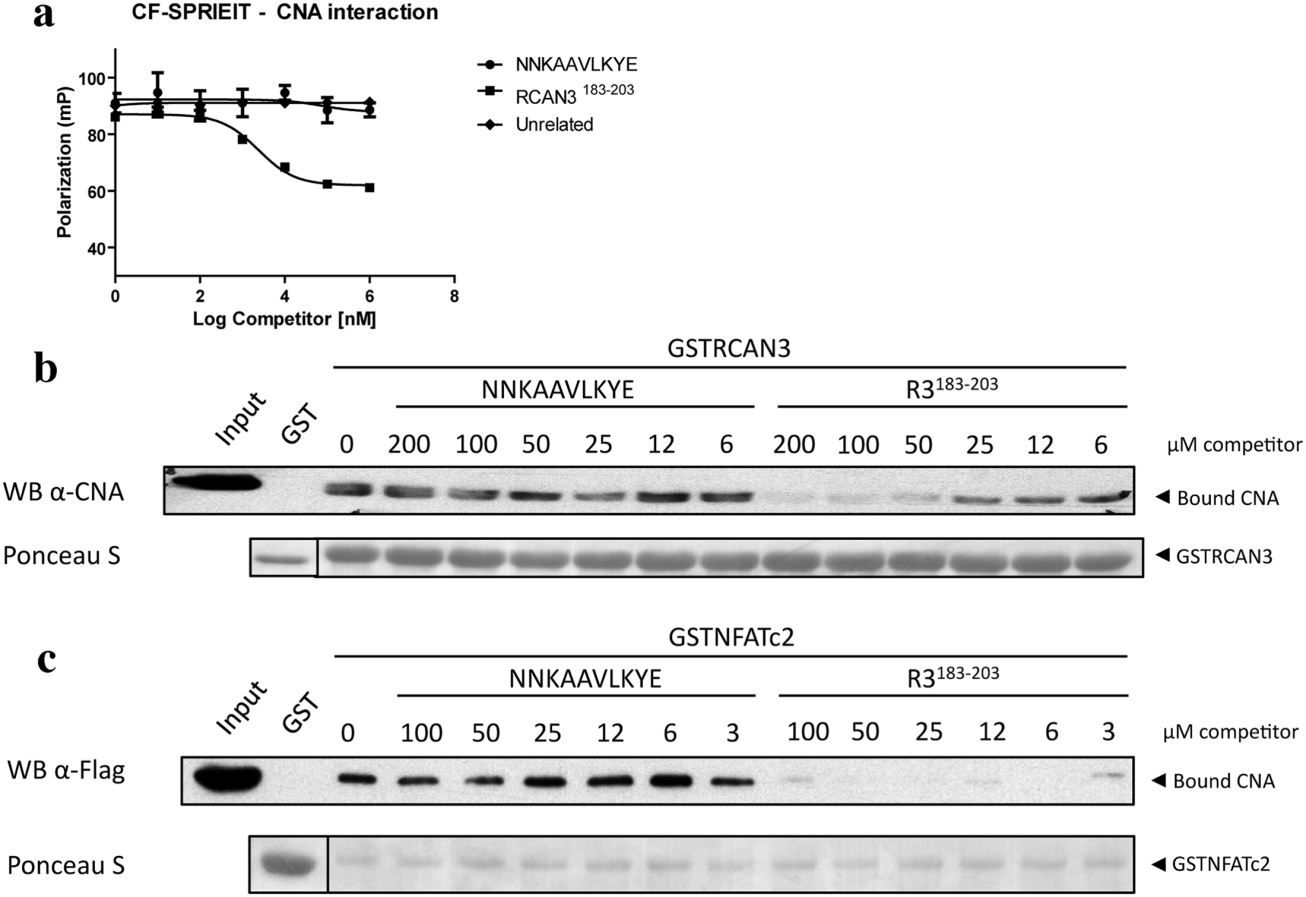
The conserved *cis*-CNA β14 strand structure does not seem to include a functional PxIxIT binding site. (**a**) CF-SPRIEIT-CNA interaction was competed with increasing amounts of the unlabeled CNA-derived peptide NNKAAVLKYE from *cis*-CNA β14 strand or an RCAN3-derived PxIxIT-containing peptide (amino acids 183–203, R3^183-203^) and assessed by fluorescence anisotropy. Anisotropic fluorescence emission values (mP) are represented as mean ±SEM of two independent experiments performed in triplicates. An unrelated peptide was included as negative control. Endogenous CNA (**b**) or Flag-CNA 2–389 (**c**) pull down assays using GST-RCAN3 (GSTR3) (**b**) or GST-NFATc2 (GSTNFATc2) (**c**) as bait and competed with increasing concentrations of *cis-*CNA-derived peptide NNKAAVLKYE or RCAN3-derived peptide (R3^183-203^). GST alone was used as negative control. Ponceau staining of the membrane shows equal GST fusion protein loading of each lane.

**Fig 7:**
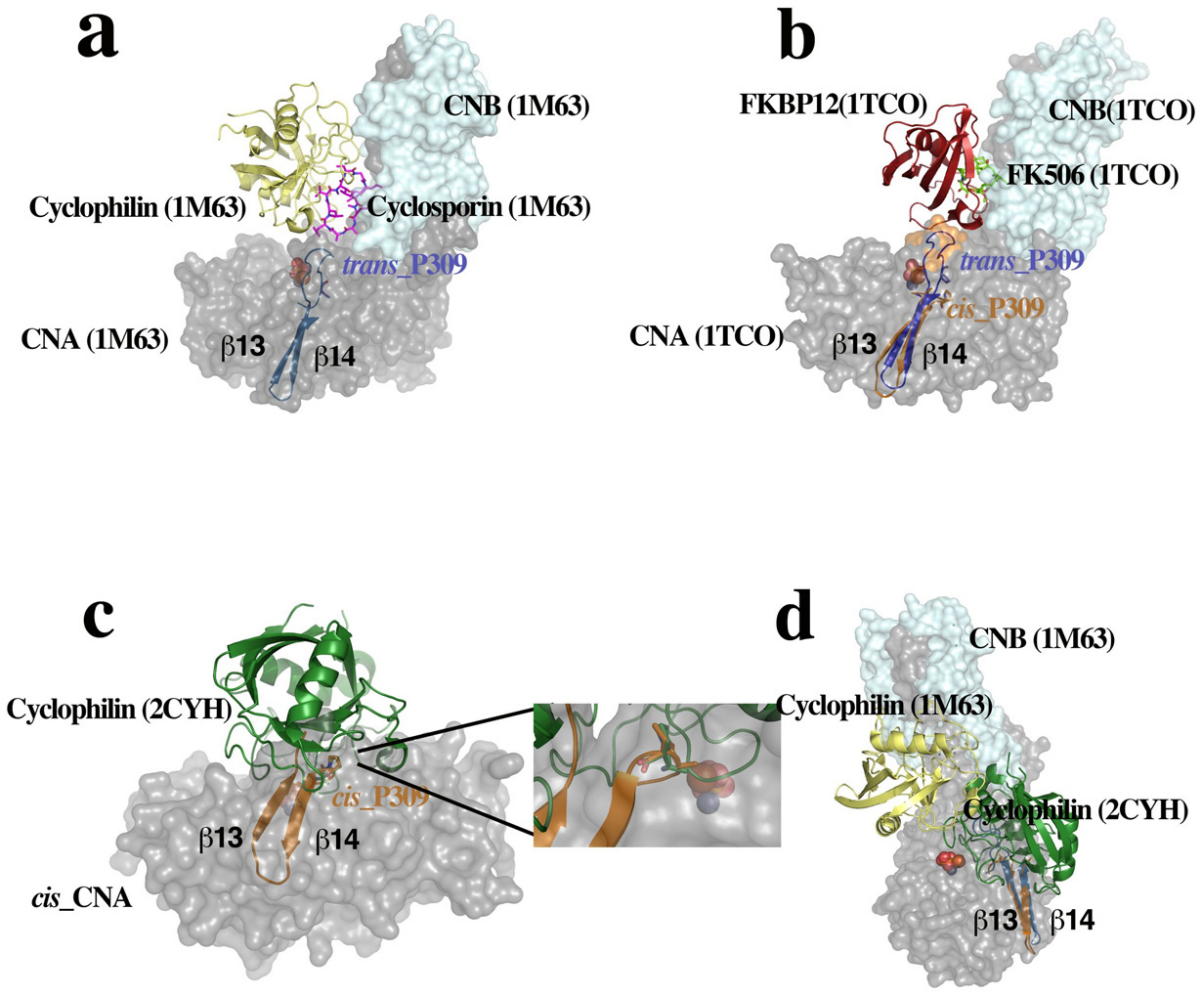
Complexes of CN with peptidyl-prolyl isomerases (PPIs) Cyclophilin A (CypA) and FKBP12. The crystal structures of CN (surface representation) in complex with (**a**) CypA and the immunosuppressant drug cyclosporine A (CsA) (PDB code 1M63) and (**b**) FKBP12 and the immunosuppressant drug FK506 (PDB code 1TCO) place both PPIs in similar locations with direct access to Pro309 if CNA adopts the *cis* conformation. The solvent accessible loop 7 in *trans-*CNA (blue) is absent in *cis*-CNA (brown), where Pro309 is placed at the tip of the β13-β14 turn. (**c**) Docking of the structure of the complex of CypA with the Ala-*cis*Pro dipeptide (PDB code 2CYH) onto *cis*-CNA by superimposition of the Ala-*cis*Pro fragments of both structures (shown in detail in the inset). (**d**) The interaction between PPIs and the catalytic domain of *cis*-CNA requires binding at sites that are close but not identical to the ones observed in the CN-Immunosuppressant-PPI ternary complexes, which can explain why CNB is dispensable for the FKBP12-CN binary complex but required for the ternary complex with FK506 [34].

In all the reported structures of catalytic domains from PPPs the central proline in the highly conserved SAPNY sequence shows the peptidyl *trans* isomer found also in the *trans*-CNA subunit in this work [21, 23, 33]. Strikingly, in the only available structure of a protein unrelated to PPP phosphatases containing a SAPNY sequence (the prokaryotic enzyme family 5-xyloglucanase, PDB codes 2JEP and 2JEQ), the peptide forms a solvent exposed turn with a central *cis*-proline that is essentially identical to the structure found in *cis*-CNA (Fig 3c). The parallel pairing of β14 strands from neighbor CNA subunits having to deviate from a symmetric two fold interaction can explain, at least in part, the presence of the alternative conformations. This supports the feasibility of the *trans-* to *cis* transition, not yet detected directly *in vivo*, when the appropriated interactions with other proteins are involved. The standard PxIxIT binding site in the *trans*-CNA subunit (sequence VMNIRQF starting with Val328), is occupied by the shifted sequence AAVLKYE (starting with Ala319) from the β14 strand of the neighbor *cis*-CNA (Figs 1b and 2b). However, the peptide NNKAAVLKYE, containing the sequence of β-14 strand of cis-CNA, does not interfere with high affinity interactions between CN and peptides or proteins containing a PxIxIT motif such a the NFATc2-derived SPRIEIT peptide in fluorescence polarization assays (Fig 6a) and the GSTRCAN3 (Fig 6b) or the GSTNFATc2 (Fig 6c) proteins in pull down assays. In contrast, the RCAN3-derived R3^183-203^ peptide, which includes a PxIxIT motif, is able to disrupt CN interaction with proteins containing a PxIxIT motif. The corresponding site in the β14 strand of the *cis*-CNA subunit (AAVLKY) interacts with the NVMNIR sequence (starting with Asn327 from the β14 strand of the neighbor *trans*-CNA subunit). The reverse c*is*- to *trans*-CNA transition might be enhanced by peptidyl-prolyl isomerase enzymes (PPIs), given the accessibility of Pro309 exposed at the molecular surface (Fig 4d). In fact, a wealth of data exists that defines CN as one of the *in vivo* targets of FKBP12 and CypA, two ubiquitous PPIs that are known to physically interact with and modulate CN even in the absence of the FK506 and CsA immunosuppressant drugs[34, 35]. Furthermore, structures of CN in complex with either FKBP12-FK506 [11, 12] or with CypA-CsA [19, 20] place both PPIs in similar locations (Fig 7a and 7b) having access to Pro309 if the *cis*-CNA conformation is adopted. Docking of the structure of the complex of CypA with the Ala-*cis*Pro dipeptide (PDB code 2CYH) onto *cis*-CNA by superimposition of the Ala-*cis*Pro fragments of both structures indicates a close interaction between CypA and the catalytic domain of CNA (Fig 7c and 7d), which can explain why CNB is dispensable for the FKBP12-CN binary complex but required for the ternary complex with FK506[34]. A prolyl isomerization process provides a rationale for the intriguing interactions of CN and PPIs, with natural products CsA and FK506 capitalizing upon these inherent interactions[34]. A similar isomerization mechanism could also operate for the mammalian target of rapamycin kinase (mTOR), which forms a ternary complex with FKBP12 and the natural product rapamycin[36]. Phosphorylation-dependent prolyl isomerization has been proposed as a molecular timer to modulate the amplitude and duration of cellular processes[37]. Complex patterns of regulation could also result from prolyl isomerization and PPIs modulating Cn.

The high sequence and structural similarities between the catalytic domains of PPPs suggests that the alternative *trans*- *cis-* conformations associated with peptidyl isomerization of the proline from the conserved SAPNY sequence observed for CNA can be common to most PPPs. In particular, interactions at the RVxF binding site of PP1 could present a similar feature to the one proposed here for the PxIxIT binding site of CN. For PPPs an increased structural versatility can help to explain how they can achieve specificity towards a large number of substrates, besides other established mechanisms [1, 4, 24]. It also provides a new vista for the development of therapeutic drugs with specificity directed towards one or the other of the two alternative conformers.

## References

[1] Roy J, Cyert MS. Cracking the phosphatase code: docking interactions determine substrate specificity. Sci Signal. 2009;2(100):re9 Epub 2009/12/10. 10.1126/scisignal.2100re9 . 19996458

[2] Shi Y. Serine/threonine phosphatases: mechanism through structure. Cell. 2009;139(3):468–84. Epub 2009/11/03. 10.1016/j.cell.2009.10.006 . 19879837

[3] Cohen PTW. Overview of protein serine/threonine phosphatases Topics in Current Genetics: protein phosphatases. 2004;5(Arino J., Alexander D.R., editors. Springer-Verlag; Berlin–Heidelberg):10.

[4] Virshup DM, Shenolikar S. From promiscuity to precision: protein phosphatases get a makeover. Mol Cell. 2009;33(5):537–45. Epub 2009/03/17. 10.1016/j.molcel.2009.02.015 . 19285938

[5] Aramburu J, Rao A, Klee CB. Calcineurin: from structure to function. Curr Top Cell Regul. 2000;36:237–95. Epub 2000/06/08. . 10842755 10.1016/s0070-2137(01)80011-x

[6] Borel JF, Feurer C, Gubler HU, Stahelin H. Biological effects of cyclosporin A: a new antilymphocytic agent. Agents Actions. 1976;6(4):468–75. Epub 1976/07/01. . 8969 10.1007/BF01973261

[7] Kino T, Hatanaka H, Hashimoto M, Nishiyama M, Goto T, Okuhara M, et al FK-506, a novel immunosuppressant isolated from a *Streptomyces*. I. Fermentation, isolation, and physico-chemical and biological characteristics. J Antibiot (Tokyo). 1987;40(9):1249–55. Epub 1987/09/01. . 2445721 10.7164/antibiotics.40.1249

[8] Liu J, Farmer JD, Lane WS, Friedman J, Weissman I, Schreiber SL. Calcineurin is a common target of cyclophilin-cyclosporin A and FKBP-FK506 complexes. Cell. 1991;66:807–15. 1715244 10.1016/0092-8674(91)90124-h

[9] Gaston RS. Chronic calcineurin inhibitor nephrotoxicity: reflections on an evolving paradigm. Clin J Am Soc Nephrol. 2009;4(12):2029–34. Epub 2009/10/24. 10.2215/CJN.03820609 . 19850771

[10] Perrino BA. Regulation of calcineurin phosphatase activity by its autoinhibitory domain. Arch Biochem Biophys. 1999;372(1):159–65. Epub 1999/11/24. 10.1006/abbi.1999.1485 . 10562429

[11] Kissinger CR, Parge HE, Knighton DR, Lewis CT, Pelletier LA, Tempczyk A, et al Crystal structures of human calcineurin and the human FKBP12-FK506-calcineurin complex. Nature. 1995;378(6557):641–4. Epub 1995/12/07. 10.1038/378641a0 . 8524402

[12] Griffith JP, Kim JL, Kim EE, Sintchak MD, Thomson JA, Fitzgibbon MJ, et al X-ray structure of calcineurin inhibited by the immunophilin-immunosuppressant FKBP12-FK506 complex. Cell. 1995;82(3):507–22. Epub 1995/08/11. . 7543369 10.1016/0092-8674(95)90439-5

[13] Wei Q, Lee EY. Mutagenesis of the L7 loop connecting beta strands 12 and 13 of calcineurin: evidence for a structural role in activity changes. Biochemistry. 1997;36(24):7418–24. Epub 1997/06/17. 10.1021/bi962703s . 9200689

[14] Xie X, Xue C, Huang W, Yu D, Wei Q. The b12-b13 loop is a key regulatory element fot the activity and properties of the catalytic domain of protein phosphatase 1 and 2B. Biol Chem. 2006;387:1461–7. 17081120 10.1515/BC.2006.183

[15] Maynes JT, Perreault KR, Cherney MM, Luu HA, James MN, Holmes CF. Crystal structure and mutagenesis of a protein phosphatase-1:calcineurin hybrid elucidate the role of the beta12-beta13 loop in inhibitor binding. J Biol Chem. 2004;279(41):43198–206. Epub 2004/07/29. 10.1074/jbc.M407184200 . 15280359

[16] Aramburu J, Yaffe MB, Lopez-Rodriguez C, Cantley LC, Hogan PG, Rao A. Affinity-driven peptide selection of an NFAT inhibitor more selective than cyclosporin A. Science. 1999;285(5436):2129–33. Epub 1999/09/25. . 10497131 10.1126/science.285.5436.2129

[17] Martinez-Martinez S, Rodriguez A, Lopez-Maderuelo MD, Ortega-Perez I, Vazquez J, Redondo JM. Blockade of NFAT activation by the second calcineurin binding site. J Biol Chem. 2006;281(10):6227–35. Epub 2006/01/13. 10.1074/jbc.M513885200 . 16407284

[18] Grigoriu S, Bond R, Cossio P, Chen JA, Ly N, Hummer G, et al The molecular mechanism of substrate engagement and immunosuppressant inhibition of calcineurin. PLoS Biol. 2013;11(2):e1001492 Epub 2013/03/08. 10.1371/journal.pbio.1001492 23468591 PMC3582496

[19] Jin L, Harrison SC. Crystal structure of human calcineurin complexed with cyclosporin A and human cyclophilin. Proc Natl Acad Sci USA. 2002;99(21):13522–6. Epub 2002/10/03. 10.1073/pnas.212504399 12357034 PMC129706

[20] Huai Q, Kim HY, Liu Y, Zhao Y, Mondragon A, Liu JO, et al Crystal structure of calcineurin-cyclophilin-cyclosporin shows common but distinct recognition of immunophilin-drug complexes. Proc Natl Acad Sci USA. 2002;99(19):12037–42. Epub 2002/09/10. 10.1073/pnas.192206699 12218175 PMC129394

[21] Li H, Zhang L, Rao A, Harrison SC, Hogan PG. Structure of calcineurin in complex with PVIVIT peptide: portrait of a low-affinity signalling interaction. J Mol Biol. 2007;369(5):1296–306. Epub 2007/05/15. 10.1016/j.jmb.2007.04.032 . 17498738

[22] Li H, Pink MD, Murphy JG, Stein A, Dell'Acqua ML, Hogan PG. Balanced interactions of calcineurin with AKAP79 regulate Ca2+-calcineurin-NFAT signaling. Nat Struct Mol Biol. 2012;19(3):337–45. Epub 2012/02/22. 10.1038/nsmb.2238 22343722 PMC3294036

[23] Takeuchi K, Roehrl MH, Sun ZY, Wagner G. Structure of the calcineurin-NFAT complex: defining a T cell activation switch using solution NMR and crystal coordinates. Structure. 2007;15(5):587–97. Epub 2007/05/16. 10.1016/j.str.2007.03.015 17502104 PMC1989110

[24] Peti W, Nairn AC, Page R. Structural basis for protein phosphatase 1 regulation and specificity. Febs J. 2013;280(2):596–611. Epub 2012/01/31. 10.1111/j.1742-4658.2012.08509.x 22284538 PMC3350600

[25] Mulero MC, Aubareda A, Orzaez M, Messeguer J, Serrano-Candelas E, Martinez-Hoyer S, et al Inhibiting the calcineurin-NFAT (nuclear factor of activated T cells) signaling pathway with a regulator of calcineurin-derived peptide without affecting general calcineurin phosphatase activity. J Biol Chem. 2009;284(14):9394–401. Epub 2009/02/05. 10.1074/jbc.M805889200 19189965 PMC2666591

[26] Battye TG, Kontogiannis L, Johnson O, Powell HR, Leslie AG. iMOSFLM: a new graphical interface for diffraction-image processing with MOSFLM. Acta Crystallogr D Biol Crystallogr. 2011;67(Pt 4):271–81. Epub 2011/04/05. 10.1107/S0907444910048675 21460445 PMC3069742

[27] Lebedev AA, Vagin AA, Murshudov GN. Model preparation in MOLREP and examples of model improvement using X-ray data. Acta Crystallogr D Biol Crystallogr. 2008;64(Pt 1):33–9. Epub 2007/12/21. 10.1107/S0907444907049839 18094465 PMC2394799

[28] Murshudov GN, Skubak P, Lebedev AA, Pannu NS, Steiner RA, Nicholls RA, et al REFMAC5 for the refinement of macromolecular crystal structures. Acta Crystallogr D Biol Crystallogr. 2011;67(Pt 4):355–67. Epub 2011/04/05. 10.1107/S0907444911001314 21460454 PMC3069751

[29] Emsley P, Cowtan K. Coot: model-building tools for molecular graphics. Acta Crystallogr D Biol Crystallogr. 2004;60(Pt 12 Pt 1):2126–32. Epub 2004/12/02. 10.1107/S0907444904019158 . 15572765

[30] Martinez-Hoyer S, Aranguren-Ibanez A, Garcia-Garcia J, Serrano-Candelas E, Vilardell J, Nunes V, et al Protein kinase CK2-dependent phosphorylation of the human Regulators of Calcineurin reveals a novel mechanism regulating the calcineurin-NFATc signaling pathway. Biochim Biophys Acta. 2013;1833(10):2311–21. Epub 2013/06/05. 10.1016/j.bbamcr.2013.05.021 . 23732701

[31] Pal D, Chakrabarti P. Cis peptide bonds in proteins: residues involved, their conformations, interactions and locations. J Mol Biol. 1999;294(1):271–88. Epub 1999/11/11. 10.1006/jmbi.1999.3217 . 10556045

[32] Chan CP, Gallis B, Blumenthal DK, Pallen CJ, Wang JH, Krebs EG. Characterization of the phosphotyrosyl protein phosphatase activity of calmodulin-dependent protein phosphatase. J Biol Chem. 1986;261(21):9890–5. . 2426255

[33] Egloff MP, Johnson DF, Moorhead G, Cohen PT, Cohen P, Barford D. Structural basis for the recognition of regulatory subunits by the catalytic subunit of protein phosphatase 1. Embo J. 1997;16(8):1876–87. Epub 1997/04/15. 10.1093/emboj/16.8.1876 9155014 PMC1169791

[34] Cardenas ME, Hemenway C, Muir RS, Ye R, Fiorentino D, Heitman J. Immunophilins interact with calcineurin in the absence of exogenous immunosuppressive ligands. Embo J. 1994;13(24):5944–57. Epub 1994/12/15. 7529175 10.1002/j.1460-2075.1994.tb06940.x PMC395570

[35] Stie J, Fox D. Calcineurin regulation in fungi and beyond. Eukaryot Cell. 2008;7(2):177–86. Epub 2007/12/11. 10.1128/EC.00326-07 18065652 PMC2238167

[36] Yang H, Rudge DG, Koos JD, Vaidialingam B, Yang HJ, Pavletich NP. mTOR kinase structure, mechanism and regulation. Nature. 2013;497(7448):217–23. Epub 2013/05/03. 10.1038/nature12122 . 23636326 PMC4512754

[37] Lu KP, Finn G, Lee TH, Nicholson LK. Prolyl cis-trans isomerization as a molecular timer. Nat Chem Biol. 2007;3(10):619–29. 10.1038/nchembio.2007.35 . 17876319

[38] Katoh K, Asimenos G, Toh H. Multiple alignment of DNA sequences with MAFFT. Methods Mol Biol. 2009;537:39–64. Epub 2009/04/21. 10.1007/978-1-59745-251-9_3 . 19378139

[39] Waterhouse AM, Procter JB, Martin DM, Clamp M, Barton GJ. Jalview Version 2—a multiple sequence alignment editor and analysis workbench. Bioinformatics. 2009;25(9):1189–91. Epub 2009/01/20. 10.1093/bioinformatics/btp033 19151095 PMC2672624

